# Application of Amyloid-Based Hybrid Membranes in Drug Delivery

**DOI:** 10.3390/polym15061444

**Published:** 2023-03-14

**Authors:** You-Ren Lai, Steven S.-S. Wang, Ti-Lun Hsu, Szu-Hui Chou, Su-Chun How, Ta-Hsien Lin

**Affiliations:** 1 Department of Chemical Engineering, National Taiwan University, Taipei 10617, Taiwan; ray110135@gmail.com (Y.-R.L.); sswang@ntu.edu.tw (S.S.-S.W.);; 2 Department of Chemical Engineering and Biotechnology, Tatung University, Taipei 104, Taiwan; 3 Laboratory of Nuclear Magnetic Resonance, Department of Medical Research, Taipei Veterans General Hospital, Taipei 11217, Taiwan; 4 Institute of Biochemistry and Molecular Biology, National Yang Ming Chiao Tung University, Taipei 11221, Taiwan

**Keywords:** amyloid, membrane, drug delivery, whey protein isolate, carboxymethyl cellulose

## Abstract

The properties of amyloid fibrils, e.g., unique structural characteristics and superior biocompatibility, make them a promising vehicle for drug delivery. Here, carboxymethyl cellulose (CMC) and whey protein isolate amyloid fibril (WPI-AF) were used to synthesize amyloid-based hybrid membranes as vehicles for the delivery of cationic and hydrophobic drugs (e.g., methylene blue (MB) and riboflavin (RF)). The CMC/WPI-AF membranes were synthesized via chemical crosslinking coupled with phase inversion. The zeta potential and scanning electron microscopy results revealed a negative charge and a pleated surface microstructure with a high content of WPI-AF. FTIR analysis showed that the CMC and WPI-AF were cross-linked via glutaraldehyde and the interacting forces between membrane and MB or RF was found to be electrostatic interaction and hydrogen bonding, respectively. Next, the in vitro drug release from membranes was monitored using UV-vis spectrophotometry. Additionally, two empirical models were used to analyze the drug release data and relevant rate constant and parameters were determined accordingly. Moreover, our results indicated that in vitro drug release rates depended on the drug–matrix interactions and transport mechanism, which could be controlled by altering the WPI-AF content in membrane. This research provides an excellent example of utilizing two-dimensional amyloid-based materials for drug delivery.

## 1. Introduction

Amyloid fibrils are highly ordered protein aggregates that are formed by the self-assembly of soluble peptides/proteins [1,2,3,4]. Amyloid fibrillar aggregates have long been considered as pathological conformers, which are associated with several diseases, e.g., Alzheimer’s disease, Parkinson’s disease, and type II diabetes [5,6,7,8]. It is widely accepted that amyloid fibril formation follows a nucleation-dependent polymerization mechanism [9,10,11,12,13]. Ample lines of evidence have suggested that both disease-related and non-disease-related proteins/peptides are capable of forming amyloid fibrils under certain conditions, highlighting that amyloid fibrillogenesis is a common property of polypeptides [5,6,14,15,16,17,18,19].

Amyloid fibrils are formed by dense packing of hydrogen bonds that contributes to the backbone–backbone interactions of the intermolecular polypeptide chains [20,21,22]. The rigidity of amyloid fibrils’ cross β-sheet core structure, which is formed through the noncovalent interactions, is considered to be responsible for the excellent mechanical properties of amyloid fibrils. Given their highly repeated and ordered architecture, amyloid fibrils have been demonstrated to exhibit the following favorable properties [23,24,25]: (1) Aggregated species with different topographical characteristics (e.g., size, shape, and morphology) and/or structures can be produced by modulating the experimental conditions (e.g., pH, stirring, temperature, salt concentration). (2) The physicochemical properties of amyloid fibrils can be modulated by altering the amino acid sequence. (3) Amyloid fibrils have nice stability against harsh environmental conditions. (4) Amyloid fibrils exhibit superior mechanical properties (e.g., stiffness/stiffness comparable to silk and steel [26]; high Young’s modulus [27] and ultimate strength [28]). (5) Amyloid fibrils can be readily functionalized with surface modifications [29,30]. (6) Amyloid fibrils show nice biodegradability and biocompatibility [31,32]

The aforesaid remarkable features foster the idea of utilizing amyloid fibrils as building blocks for bioinspired materials, and facilitate the exploitation of amyloid fibrils as a class of advanced nanomaterials/bio-nanomaterials [33]. Increasing evidence has revealed the potential applications of amyloid-based materials in the fields of electronics, biomedicine, biochemical engineering, reaction engineering, and separation engineering [34,35,36,37]. The complex networks of filaments, gels, and membranes made from protein amyloid fibrils have been demonstrated to be suitable for encapsulating and/or immobilizing enzymes and drugs [38,39]. However, there have been only limited examples and research reports focusing on the application of amyloid fibril-based materials in drug delivery, which warrant further investigation.

In this work, amyloid-based hybrid membranes composed of carboxymethyl cellulose (CMC) and whey protein isolate amyloid fibril (WPI-AF) were prepared and used as drug release vehicles for the delivery of cationic (e.g., methylene blue (MB) and hydrophobic (e.g., riboflavin (RF)) drugs. The CMC/WPI-AF membranes were first synthesized via chemical crosslinking coupled with phase inversion. Apart from characterizing the zeta potential and microstructure of the CMC/WPI-AF membranes, FTIR was employed to analyze the governing interactions between membranes and MB or RF. Finally, the in vitro passive release of MB or RF from the membranes was monitored and the release kinetic data were fitted against two empirical models to obtain the key model parameters and gain some insights into the molecular mechanism of drug release. We believe this research provides an excellent example of applying the amyloid-based materials for drug delivery.

## 2. Materials and Methods

### 2.1. Materials

The whey protein isolate (~90% content) used in this study was kindly supplied by the Food Industry Research and Development Institute (FIRDI), Taiwan. The composition and key properties of WPI are listed in Table S1. The carboxymethyl cellulose sodium salt with a degree of substitution of 0.7 and the glutaraldehyde crosslinker were obtained from Sigma-Aldrich. The potassium phosphate monobasic and sodium chloride were also both acquired from Sigma-Aldrich. Moreover, the methylene blue (MB) and riboflavin (RF) were purchased from Acros Organics and Sigma-Aldrich, respectively (see Figure S1 for the chemical structures of the MB and RF).

### 2.2. Amyloid Fibril Formation of the Whey Protein Isolate (WPI)

The WPI solution was prepared by dissolving 600 mg of WPI powder in 15 mL of deionized water. The pH value of the WPI solution was then altered to achieve an acidic condition (pH 2.0) using 6 N hydrogen chloride (HCl), before it was mixed well by means of vortexing. Afterward, the WPI solution was incubated at 80 °C and 600 rpm stirring for a day to yield a solution containing WPI amyloid fibril (WPI-AF). Finally, the WPI-AF solution was stored at 4 °C until required for fabricating the hybrid membrane.

### 2.3. Fabrication of the Carboxymethyl Cellulose/Whey Protein Isolate Amyloid Fibril (CMC/WPI-AF) Membrane

Blend solutions containing WPI-AF and CMC were prepared with different CMC:WPI-AF mass ratios of 1:1 and 1:2 at a pH of 2.0. Next, 10 mg/mL of glutaraldehyde was added as a crosslinker to the CMC/WPI-AF blend solutions, which were then mixed at 1200 rpm at room temperature via a magnetic stirrer for a day. Subsequently, 2 mL of the CMC/WPI-AF mixture solutions were added in a dropwise fashion to a glass plate (length × width = 6 cm × 6 cm) and kept at room temperature for three days. Afterward, the CMC/WPI-AF cast on the glass plate was soaked in ethanol in order to synthesize the CMC/WPI-AF membranes. Finally, the CMC/WPI-AF membranes were wiped with filter paper to remove any excess ethanol and then stored in Petri dishes for the subsequent experiments and characteristic analysis.

### 2.4. Thioflavin T (ThT) Binding Assay

The ThT dye powder was first weighed and then dissolved in ethanol to prepare the ThT stock solution. The actual concentration of the ThT stock solution was determined to be 189 μM, using a Cary 50 UV-Vis Spectrophotometer (Varian, Palo Alto, CA, USA), and also spectrophotometrically, using the extinction coefficient of 26,600 M cm^−1^ at 416 nm. Next, the ThT working solution was prepared by diluting the ThT stock solution to a final concentration of 10 μM using PB buffer. Afterward, 20 μL aliquots of the sample solutions were mixed with 480 μL of the ThT working solution, and 350 μL of the mixture solutions were then pipetted into a 1 cm light path quartz cuvette. The ThT fluorescence spectra of the samples were obtained using a Cary Eclipse Fluorescence Spectrophotometer (Varian, USA) with the excitation wavelength set at 440 nm and the emission wavelength set at a range of 450 to 550 nm. Finally, the data of ThT fluorescence intensity at 485 nm were plotted against incubation time and fitted by the Boltzmann sigmoidal equation shown below [40,41]:

(1) $$F=F_{f}+\frac{F_{i}-F_{f}}{1+\exp^{(t-t_{1/2\;})\;/\;\tau }}$$

where F is the ThT fluorescence intensity (at 485 nm); F_i_ and F_f_ are the ThT fluorescence intensities at 485 nm at the initial and final times; t_1/2_ is the time required to reach half of the elongation phase; and τ denotes the elongation time constant.

### 2.5. Transmission Electron Microscopy (TEM)

10 µL of the sample solutions were placed on a 200 mesh carbon-stabilized/formvar-coated grid for 20 s, and the excess sample solutions on the grid were thoroughly removed using filter paper. Next, the samples remaining on the grid were negatively stained using 10 µL of 1% (*w/v*) uranyl acetate solution for 90 s and left to air-dry for 30 min. Afterward, the sample grids were examined and photographed using a Hitachi H-7650 Transmission Electron Microscope with a Gantan model 782 CCD Camera (Japan) at an accelerating voltage of 75 kV.

### 2.6. Zeta-Potential Measurements

The zeta-potential measurements were performed using a Malvern Zetasizer (Nano ZS, Malvern Instruments, UK) with a folded capillary zeta cell (DTS1070, Malvern Instruments, UK). The measurement parameters for each sample were set as follows: 60 s equilibrium duration, 10 s measurement duration, 10 measurements, and water as the dispersant/solvent at room temperature. The mean ± standard deviation of the zeta-potential value of samples was evaluated using Malvern Zetasizer software.

### 2.7. Fourier Transform Infrared (FT-IR) Spectroscopy

The functional groups of the CMC/WPI-AF membranes with different CMC:WPI-AF mass ratios and loading drugs (e.g., MB and RF) were investigated using a Perkin Elmer Spectrum 1000 FT-IR Spectrometer. Prior to the measurements, all the membranes were dehydrated via air-drying at 100 °C for a day and then grind-mixed following a 1:100 weight ratio with potassium bromide (KBr) powder. Next, the sample powder was compressed into tablets. Afterward, the FT-IR spectra of the sample tablets were obtained by means of an FT-IR spectrometer using a wavenumber range of 450–4000 cm^−1^.

### 2.8. Scanning Electron Microscopy (SEM)

The surface microstructures of the CMC/WPI-AF membranes with different CMC:WPI-AF mass ratios were examined using a scanning electron microscope (FEI Inspect S; FEI, USA). In a typical experiment, the CMC/WPI-AF membranes were dehydrated via air-drying at 100 °C for a day. Next, the surface of each membrane was coated with platinum using a metal coating device (VD MSP-1S) under vacuum conditions. Finally, the surface microstructure of each membrane coated with platinum was examined and imaged by means of SEM.

### 2.9. Absorption of the Drug in the CMC/WPI-AF Membrane

MB, as a model of the cationic drug, and RF, as a model of the hydrophobic drug, were loaded into the CMC/WPI-AF membranes. First, stock solutions of 0.1 M MB and 0.1 M RF were prepared by dissolving appropriate amounts of MB and RF in PBS buffer and 0.5 N NaOH solution, respectively. Next, immersion solutions of 0.02 M MB and 0.01 M RF were produced by diluting the stock solutions with PBS buffer. To allow each drug to be loaded into the CMC/WPI-AF membranes, the membranes were cut into 4 cm^2^ (length × width = 2 cm × 2 cm) pieces and then immersed in the MB and RF immersion solutions until absorption equilibrium was achieved, thereby obtaining both MB-loaded CMC/WPI-AF membranes and RF-loaded CMC/WPI-AF membranes. The concentrations of the drugs (MB or RF) loaded into the CMC/WPI-AF membranes were determined using a UV-Vis spectrophotometer (Thermo Fisher, Waltham, MA, USA) and the pre-calibration curves (see Figure S2). Finally, to assess the ability of the CMC/WPI-AF membranes with different CMC:WPI-AF mass ratios with regard to MB and RF loading, the two principal parameters, namely, the encapsulation efficiency (EE%) and the loading capacity (LC%), were determined. The evaluation of the two parameters was performed as expressed in Equations (2) and (3), respectively:

(2) $$\mathrm{EE}\;(\%)=\frac{\mathrm{weight}\;\mathrm{of}\;\mathrm{drug}\;\mathrm{loaded}\;\mathrm{in}\;\mathrm{the}\;\mathrm{membrane}\;(g)}{\mathrm{weight}\;\mathrm{of}\;\mathrm{drug}\;\mathrm{in}\;\mathrm{the}\;\mathrm{solution}\;(g)}\times 100\%$$

(3) $$\mathrm{LC}\;(\%)=\frac{\mathrm{weight}\;\mathrm{of}\;\mathrm{drug}\;\mathrm{loaded}\;\mathrm{in}\;\mathrm{the}\;\mathrm{membrane}\;(g)}{\mathrm{total}\;\mathrm{weight}\;\mathrm{of}\;\mathrm{membrane}\;(g)}\times 100\%$$

### 2.10. In Vitro Passive Drug Delivery Studies

The in vitro drug release of the MB and RF from the CMC/WPI-AF membranes was performed in the form of passive drug delivery. The procedure for the passive drug delivery was as described in a previous study [42], albeit with minor modifications. First, the MB-loaded CMC/WPI-AF membranes and RF-loaded CMC/WPI-AF membranes were immersed in PBS buffer solution (pH 7.4) at room temperature. Next, the MB and RF release experiments were conducted in dark vessels for 3 h and 0.5 h, respectively. During the passive drug release kinetics, a UV-vis spectrophotometer (Thermo Fisher, USA) was utilized to monitor the absorbance of the MB and the RF at a wavelength of ~665 nm and ~445 nm at different time points. The percentage of drugs released was as expressed in Equation (4):

(4) $$\mathrm{Drug}\;\mathrm{released}\;(\%)=\frac{{[\mathrm{Drug}]\;}_{\mathrm{released}}}{{[\mathrm{Drug}]\;}_{\mathrm{total}}}\times 100\%$$

where [Drug]_released_ was the drug released concentration (mM) and [Drug]_total_ was the total drug concentration in the CMC/WPI-AF membrane.

The relevant kinetic parameters of the passive drug delivery were further estimated using both the first-order model (Equation (5)) and the Korsmeyer–Peppas model (Equation (6)), based on the gathered experimental data:

(5) $$\frac{M_{t}}{M_{\infty }}=1-e^{-k_{f}\;t}$$

(6) $$\frac{M_{t}}{M_{\infty }}=k_{p}\cdot {\;t\;}^{n},\;\frac{M_{t}}{M_{\infty }}<0.6$$

where M_∞_ was the mass of the drug released at an infinite time; M_t_ was the mass of the drug released at time t; and k_f_, k_p_, and n were the representative of the first-order rate constant, a parameter corresponding to the structural and geometric characteristics of the dosage form, and a diffusional exponent corresponding to the release mechanism, respectively.

### 2.11. Statistical Analysis

The data obtained from n independent measurements were calculated as the mean ± standard deviation. A statistically significant difference was determined by referring to the *p* ≤ 0.05 obtained using Student’s *t*-test on n independent measurements.

## 3. Results and Discussion

### 3.1. Formation and Characterization of the WPI-AF

**Figure 1:**
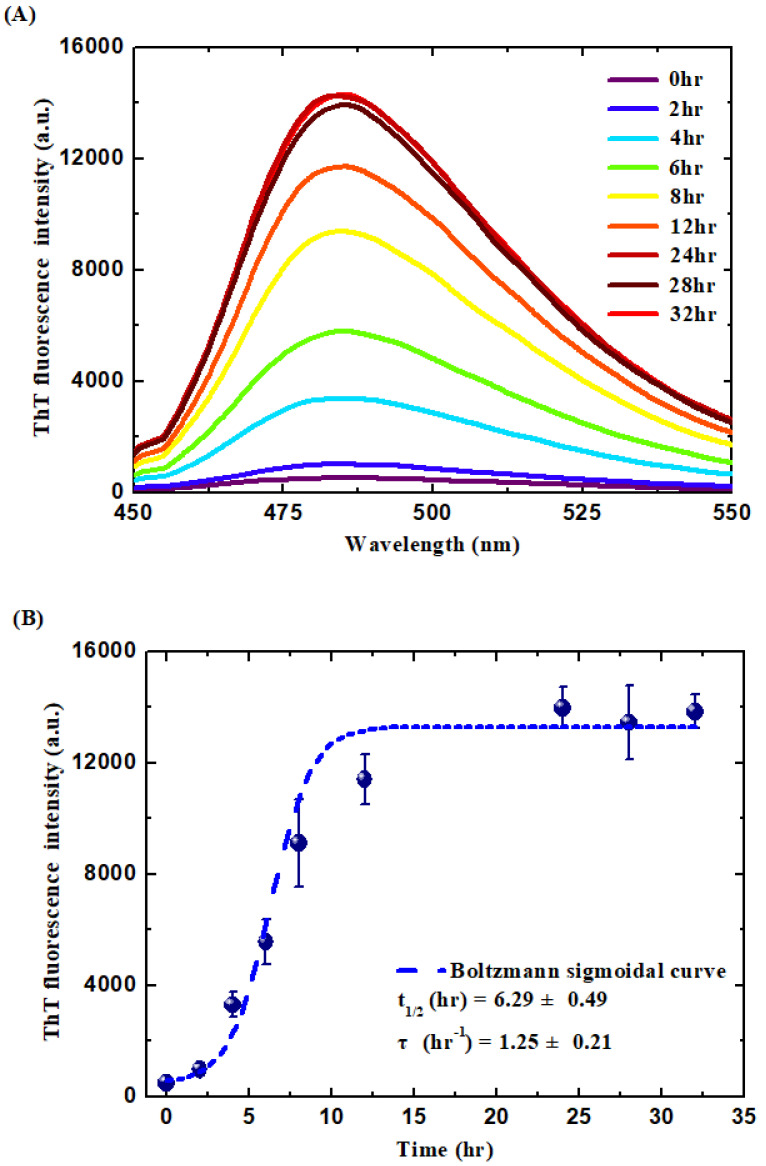
Formation of whey protein isolate amyloid fibrils as revealed by ThT binding assay. (**A**) ThT fluorescence spectra of WPI-AF at different incubation times. (**B**) ThT fluorescence intensity of WPI-AF as a function of incubation time. Boltzmann sigmoidal curve was employed to fit against the ThT fluorescence data. (The excitation wavelength = 440 nm, and the emission wavelength = 440–490 nm).

**Figure 2:**
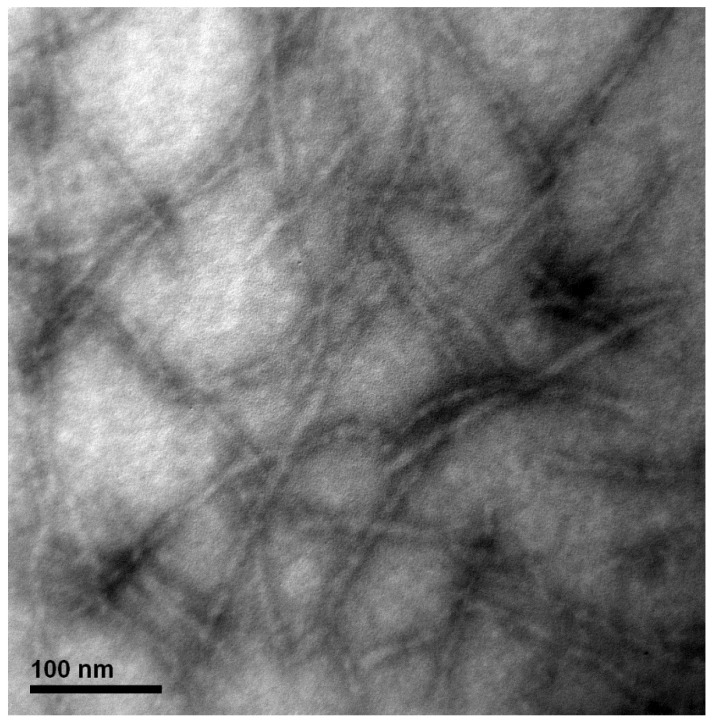
Transmission electron (TEM) micrograph of the negatively stained whey protein isolate amyloid fibrils. (The magnification = 200k×; scale bar = 100 nm).

WPI is composed of a variety of proteins, including β-lactoglobulin (β-Lg), α-lactalbumin (α-La), and bovine serum albumin (BSA), which account for 51%, 19%, and 6% of its composition, respectively [43]. To form WPI-AF via the amyloidogenesis process, the acidic (pH 2.0) WPI solution was incubated at 80 °C with 600 rpm of magnetic stirring for 24 h. Next, as the characteristics of the fluorescence emission following the addition of the ThT molecules were specifically binding on the anti-β sheet structure of the amyloid fibril [44], the fibrillation kinetic of the WPI-AF formation was monitored by means of a ThT binding assay. Figure 1A presents the ThT fluorescence spectra of the WPI-AF at 0 h and 24 h of incubation. As shown in the ThT fluorescence spectra, no significant characteristic peak was observed at 0 h of incubation time, which indicates that the WPI had not yet been induced to form the amyloid fibril. However, as the incubation time increased, there appeared an apparent characteristic peak at ~485 nm, indicating the formation of WPI-AF [44,45]. Furthermore, the ThT fluorescence intensity of the WPI-AF was detected on the basis of the incubation time. The results concerning the WPI fibrillation kinetics were consistent with the Boltzmann sigmoidal curve (see Figure 1B), which suggests that the fibrillogenesis process of the WPI was nucleation-dependent [41,44]. The nucleation-dependent amyloid fibrillogenesis process involves three phases: nucleation, elongation, and stationary [40,44]. Within the initial ~2 h, the WPI monomers aggregated to form oligomer nuclei during the nucleation phase. Next, the oligomer nuclei aggregated into protofibrils during the process of elongation from ~2 h to 12 h, before eventually entering the stationary phase to form mature fibrils after ~12 h. Moreover, the time required to reach half of the elongation phase (t_1/2_) and the elongation time constant (τ) were determined by analyzing the kinetics of the WPI-AF formation via the Boltzmann sigmoidal curve (see Figure 1B). The morphology of the WPI-AF was shown to comprise fibrillar aggregates with a high aspect ratio in the TEM micrograph (see Figure 2), which was similar to the morphology of previously reported WPI-AF [43,46]. The ThT binding assay and TEM results revealed that the WPI successfully formed amyloid fibril from the native protein.

### 3.2. Synthetic Mechanism of the CMC/WPI-AF Membranes

**Figure 3:**
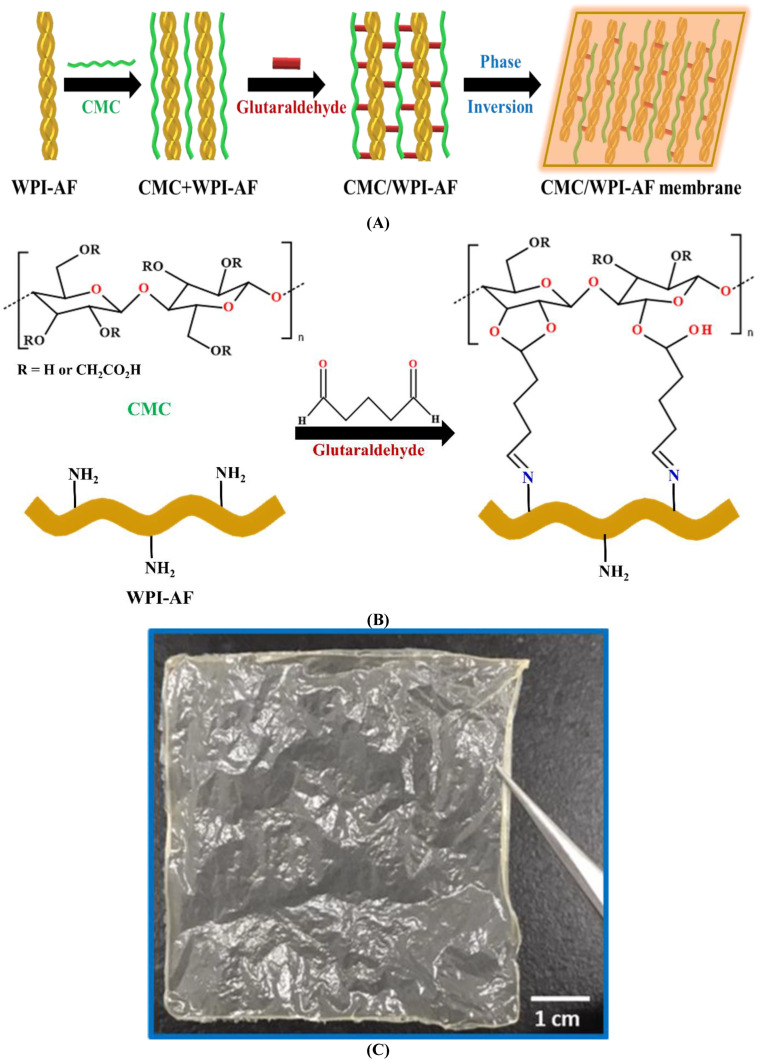
(**A**) Schematic diagram of the synthesis strategy for CMC/WPI-AF membranes. (**B**) Cross-linking of WPI-AF and CMC using glutaraldehyde. (**C**) Physical appearance of CMC/WPI-AF membrane with a CMC:WPI-AF mass ratio of 1:1. (Scale bar = 1 cm).

A schematic diagram of the synthesis strategy of the CMC/WPI-AF membranes is shown in Figure 3A. First, the WPI-AF and CMC were chemically crosslinked to form composites with interconnected networks. Within these composites, the amino and hydroxyl groups on the WPI-AF and CMC molecular chains could react with the aldehyde group of the glutaraldehyde to produce chemical crosslinking [47]. The detailed crosslinking reaction is presented in Figure 3B. In this regard, the three possible chemical crosslinking reactions are as follows. First, the substituent (amino group) of the WPI-AF could react with the aldehyde group of the glutaraldehyde to form imine-type bonds. Second, the single hydroxyl group of the CMC could react with the aldehyde group of the glutaraldehyde to form semiacetal. Third, the two hydroxyl groups of the CMC could react with the aldehyde group of the glutaraldehyde to form acetal-type rings [47]. Next, the CMC/WPI-AF membranes were synthesized by means of a phase inversion-based immersion precipitation method. With this method, a non-solvent induces the phase inversion of the polymer solution, resulting in the precipitation of the polymer species to form a membrane [48]. The chemically crosslinked CMC/WPI-AF blend solution was then cast on a glass platform and immersed in a precipitation bath containing ethanol. During this immersion process, the solvent in the CMC/WPI-AF blend solution and the non-solvent in the precipitation bath were exchanged through mass transfer, resulting in the CMC/WPI-AF membranes being synthesized via phase inversion. Figure 3C shows the CMC/WPI-AF membranes to be yellow in color, indicating that the WPI-AF are crosslinked with the CMC via the glutaraldehyde crosslinker. Furthermore, the physical appearance of the CMC/WPI-AF membrane maintained its integrity in the PBS buffer. Conversely, the membrane prepared using CMC alone disintegrated in the PBS buffer, thereby indicating that CMC cannot crosslink on its own.

### 3.3. Zeta Potential Properties of the CMC/WPI-AF Membranes

**Figure 4:**
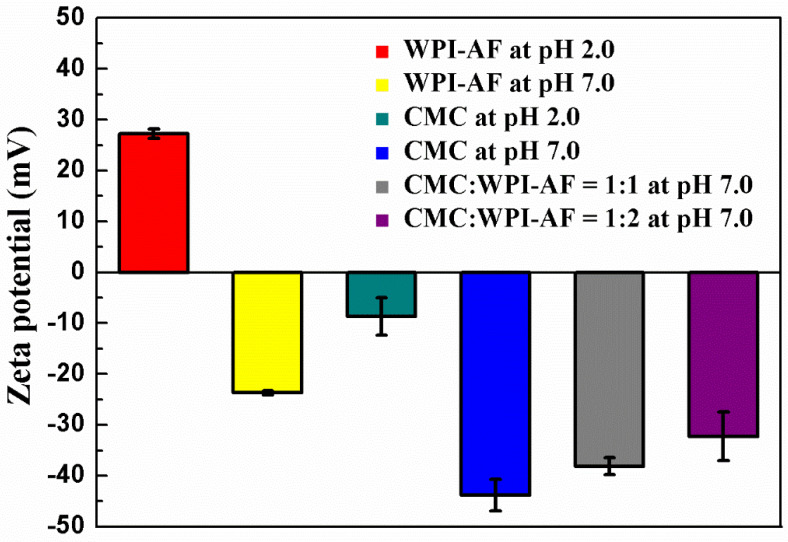
Zeta potentials of WPI-AF, CMC, and CMC/WPI-AF with different mass ratios.

As the pH value of the solvent affects the charged functional groups on the surface of the WPI-AF and CMC, the zeta potentials of the WPI-AF, CMC, and CMC/WPI-AF membranes were analyzed (see Figure 4). As the isoelectric point (pI) of the WPI was ~5.2, the zeta potential of the WPI-AF in an acidic condition (pH 2.0) was positive (27.27 ± 0.91 mV). However, when adjusted to a pH value higher than the pI, the zeta potential of the WPI-AF changed from positive to negative. This change in the zeta potential was attributed to the increase in the amount of negatively charged carboxyl groups (-COO^−^) within the protein and the neutralization of the positively charged amino groups (-NH_3_^+^) [49]. As depicted in Figure 4, the zeta potential value of the CMC was negative in both the acidic and neutral environments, and it can be observed that the amount of negative charges in the CMC increased as the pH increased, which is similar to previous findings [49,50]. These zeta potential results confirm that the CMC and WPI-AF were not only cross-linked by chemical bonds but also exhibited electrostatic interactions. Notably, the CMC/WPI-AF membranes with different CMC:WPI-AF mass ratios had zeta potentials more negative than −30 mV in a neutral environment, indicating high negative charge and stability [51].

### 3.4. Surface Microstructure Characterization of the CMC/WPI-AF Membranes

**Figure 5:**
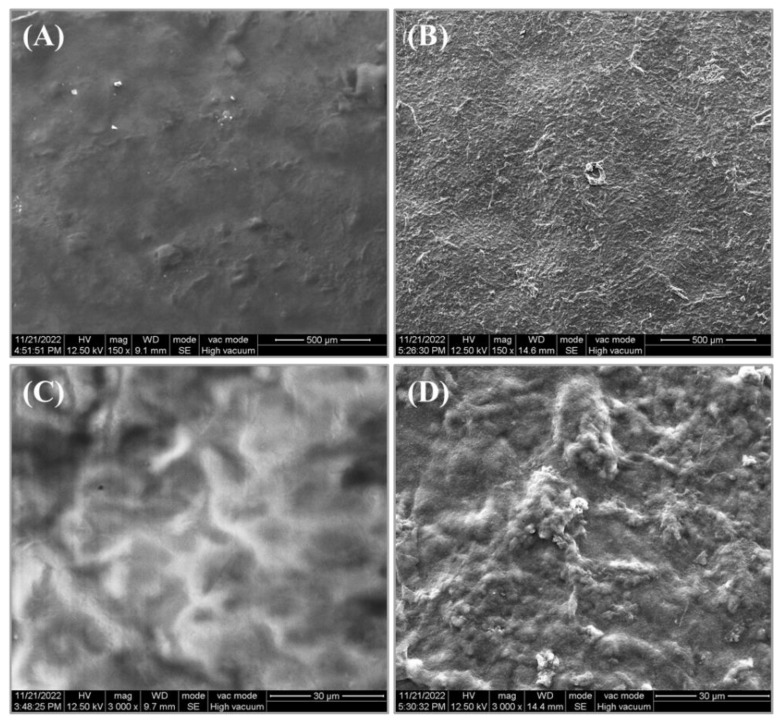
Surface microstructure of CMC/WPI-AF membranes as revealed by scanning electron microscopy (SEM). SEM images of CMC/WPI-AF membranes with CMC:WPI-AF mass ratios of 1:1 (**A**,**C**) and 1:2 (**B**,**D**). (**A**,**B**): magnification = 150×; scale bar = 500 μm; (**C**,**D**): magnification = 3000×; scale bar = 30 μm).

SEM has been widely used to characterize the surface microstructures of membranes. In this study, the samples of CMC/WPI-AF membranes were first air-dried at room temperature, and then the surface microstructures of the membranes were examined via SEM. As shown in the SEM images, there were differences in the surface microstructures of the CMC/WPI-AF membranes synthesized with different CMC:WPI-AF mass ratios (Figure 5A,B). More specifically, the surface microstructure of the membrane synthesized with a CMC:WPI-AF mass ratio of 1:1 were found to be dense and smooth at high magnifications (Figure 5C). When compared with the membrane with a CMC:WPI-AF mass ratio of 1:1, the surface microstructure of the membrane synthesized with a CMC:WPI-AF mass ratio of 1:2 showed pleated bulges at high magnifications (Figure 5D). These results suggest that CMC/WPI-AF membranes with a high content of WPI-AF exhibited surface structure of more corrugated protrusions, which may contribute to increasing their surface area [52]. We speculate that a large amount of WPI-AF was deposited and stacked on the membrane surface after the phase inversion, which resulted in apparent pleat bulges on the membrane surface.

### 3.5. FT-IR Analysis of the CMC/WPI-AF Membranes

**Figure 6:**
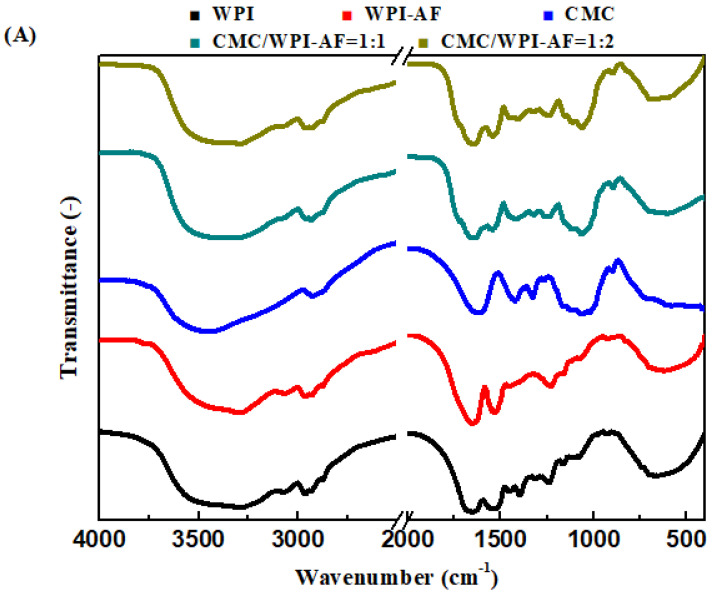

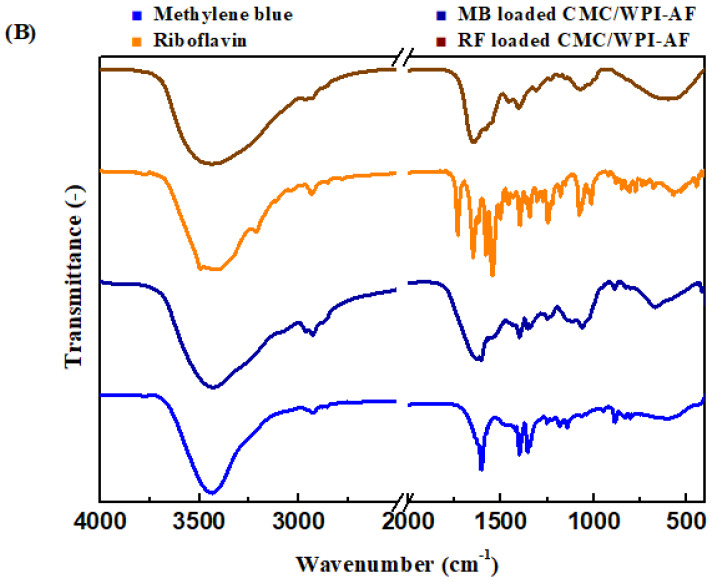
(**A**) Fourier transform infrared (FTIR) spectra of WPI, WPI-AF, CMC, and CMC/WPI-AF membranes. (**B**) FTIR spectra of methylene blue, riboflavin, MB-loaded CMC/WPI-AF membranes, and RF-loaded CMC/WPI-AF membranes.

To verify whether the crosslinking of the CMC and WPI-AF following the addition of glutaraldehyde to the solutions containing the CMC/WPI-AF samples was successful, an FT-IR spectroscopy analysis was conducted on the samples with CMC:WPI-AF mass ratios of 1:1 and 1:2. Figure 6A presents the FT-IR spectra of the native WPI, WPI-AF, CMC, and CMC/WPI-AF membranes. The secondary structure of the protein is known to be associated with a specific region of amine I (1625–1750 cm^−1^) and amine II (1475–1575 cm^−1^), which are related in the C=O stretching and N-H stretching of the peptide linkage in the protein [46,53]. The FT-IR spectra of the native WPI showed an amide I band at 1654 cm^−1^, which was downshifted to 1643 cm^−1^ following incubation at 80 ◦C and pH 2.0, thereby indicating the formation of WPI-AF concomitant with an increase in the β-sheet structure [54]. Figure 6A illustrates the characteristic peaks of the CMC at 1111 cm^−1^ and 1160 cm^−1^, which were attributed to the stretching vibration of the C–OH groups. Furthermore, the strong peaks at 1616 cm^−1^ and 1420 cm^−1^ were attributed to the carboxylic group’s asymmetric and symmetric stretching vibration [55].

In the FT-IR spectra of the CMC/WPI-AF membranes, all the characteristic peaks of the WPI-AF and CMC were observed. Due to the crosslinking of the WPI-AF and CMC via glutaraldehyde, a shift in the characteristic peaks and the formation of new peaks occurred. First, the amide II band that emerged at 1538 cm^−1^ in the FT-IR spectrum of the WPI-AF shifted to that observed at 1543 cm^−1^ in the CMC/WPI-AF membranes, indicating that the amide groups of WPI-AF can react with the carbonyl groups of glutaraldehyde to form imine group [56]. Second, the new peak seen at 1711 cm^−1^ in the FT-IR spectrum of the CMC/WPI-AF membranes was mainly associated with C=O stretching in the pendant aldehyde [47]. Third, the region of the peaks seen at ~1700–1585 cm^−1^ in the CMC/WPI-AF membranes was broader than that observed in relation to the WPI-AF and CMC, meaning that the peaks corresponding to the C=O stretching (in the amine I of the WPI-AF and the carboxylic group of the CMC) and C=N stretching (in the formed Schiff bases) overlapped [57]. These findings support the proposed crosslinking mechanism between CMC and WPI-AF, as shown in Figure 3B.

Following the drug absorption into the CMC/WPI-AF membranes, the interactions between the drugs (MB or RF) and the CMC/WPI-AF membranes were further revealed in the FT-IR spectrum (see Figure 6B). Following the absorption of the MB into the CMC/WPI-AF membranes, the peaks at 1604 cm^−1^ and 1487 cm^−1^ corresponding to the C–N and C–C bonds of the aromatic rings of the MB shifted and vanished, respectively, suggesting an interaction between the MB and membrane involving π–π interaction [58,59]. Moreover, the downshifting of the peak at 1402 cm^−1^ associated with the positive charge of nitrogen on the MB indicated an electrostatic interaction between the MB and membrane [58]. On the other hand, after the RF had absorbed into the CMC/WPI-AF membranes, the characteristic peak of the CMC/WPI-AF membrane shifted from 1627 cm^−1^ to 1640 cm^−1^. Moreover, the peaks seen at 3100–3600 cm^−1^ corresponding to the O–H and N–H stretching of the RF vanished. These peak shifts and disappearances indicated that the carboxyl groups of the CMC/WPI-AF membranes interacted with the N–H and O–H groups of the RF via hydrogen bonding [42,60]. Furthermore, the peak at 1700 cm^−1^ corresponding to the carbonyl stretching of the isoalloxazine ring of the RF disappeared after the RF had been absorbed into the CMC/WPI-AF membranes, implying that the RF was successfully absorbed and encapsulated in the membranes [61].

### 3.6. EE and LC of the Drugs on the CMC/WPI-AF Membranes

Table 1 shows that the EE% and LC% of the CMC/WPI-AF membranes were increased with an increasing amount of WPI-AF in the membranes. The possible reason for this is that the WPI-AF possessed the characteristics of a negative charge (see Figure 4) and a hydrophobic pocket mediated by aromatic residues [62,63]. Moreover, previous studies have shown that amyloid fibril can provide multiple binding sites [42,64], thereby effectively improving the EE% and LC% of CMC/WPI-AF membranes in terms of the loading of cationic and hydrophobic drugs. Furthermore, with regard to the microstructures (see Figure 5), as the amount of WPI-AF increased, the surface microstructures of the membranes exhibited more corrugated protrusions, which provided a larger surface area for the absorption drugs on the membrane surface [52]. Yet, it can be observed that the CMC/WPI-AF membranes had higher EE% and LC% in relation to the loading of cationic drugs than hydrophobic drugs. The possible reason for this, as derived from our zeta-potential and FT-IR results, may be that the CMC in the membranes can also interact with MB via electrostatic interaction. Therefore, the presence of WPI-AF and CMC in the membranes can have a synergic effect on the absorption of cationic drugs. In addition, previous literature results have shown that drugs are more adsorbable when interacting with substrate surfaces through electrostatic interactions than hydrogen bonding [65].

### 3.7. In Vitro Passive Drug Release of MB and RF from the CMC/WPI-AF Membranes

**Figure 7:**
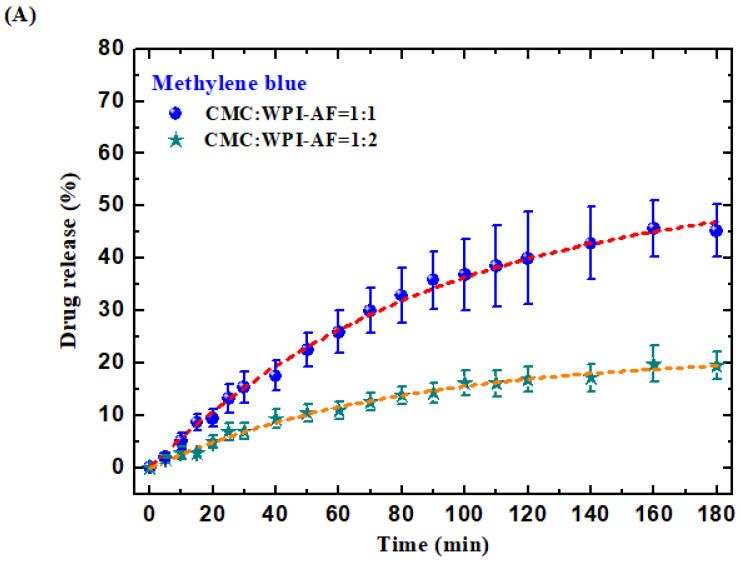

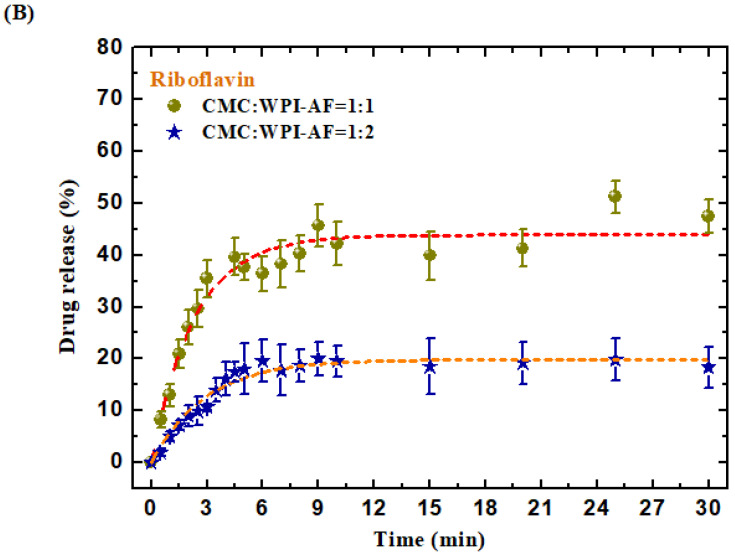
In vitro passive drug release curves of drugs from the CMC/WPI-AF membranes with different CMC:WPI-AF mass ratios ((**A**) methylene blue and (**B**) riboflavin). The CMC:WPI-AF mass ratios used were 1:1 and 1:2.

To explore the in vitro passive release of the drugs from the CMC/WPI-AF membranes, the drug release procedure was conducted in PBS at pH 7.4. Given that the release behavior of a drug in a matrix-type drug delivery system is associated with the drug-matrix interaction and the diffusion of the drug within the matrix material [66,67], our drug release condition included different types of drugs (i.e., MB and RF) and different compositions of membranes (i.e., CMC:WPI-AF mass ratios of 1:1 and 1:2). The release profiles of the MB and RF from the membrane samples with CMC:WPI-AF mass ratios of 1:1 and 1:2 were obtained by plotting the cumulative drug release percentages as a time function, as shown in Figure 7A and 7B, respectively. Then, a nonlinear regression analysis was performed to fit the MB and RF release kinetics data via the utilized empirical models, namely the first-order and Korsmeyer–Peppas models (as shown in Equations (5) and (6)), to determine the corresponding kinetic parameters and coefficients of determination (R^2^). Notably, the first-order model, which was used to describe the burst-release phenomenon, and the Korsmeyer-Peppas model, which was used to distinguish the different release mechanisms, were selected to predict the MB and RF release rate constants and decipher the diffusion behavior of the MB and RF [68,69].

According to the drug release results (as shown in Figure 7), the percentage of MB released from the CMC/WPI-AF membranes leveled off within 180 min, while the percentage of RF released from the CMC/WPI-AF membranes leveled off within 30 min. In addition, it should be noted that the percentages of MB and RF released from the membranes were reduced by increasing the WPI-AF content in the CMC/WPI-AF membranes. Concerning the different drug release behaviors upon drug release from each group, we further quantitatively analyzed the drug release results using the first-order model. As shown in Table 2, the data concerning the MB and RF released from the CMC/WPI-AF membranes adequately fit the first-order model, in which the R^2^ values for all the release curves were determined to be greater than 0.95. The fitting results of the first-order model revealed the following. First, the mass of the drug released at infinite time from the membrane was CMC:WPI-AF = 1:1 > CMC:WPI-AF = 1:2. Second, the magnitude of the first-order rate constant (k_f_) of the RF release was much larger than that of the MB release. Third, the magnitude of the first-order rate constant for the release of RF from the membrane with CMC:WPI-AF at 1:1 was found to be larger than that from the membrane with CMC:WPI-AF at 1:2, whereas the values of k_f_ (or magnitude of the first-order rate constant) for the release of MB from both membranes (CMC:WPI-AF = 1:1 and CMC:WPI-AF = 1:2) were found to be very close.

To examine the mechanism of MB and RF release from the CMC/WPI-AF membranes, the Korsmeyer–Peppas model was used to fit the drug release kinetics data. The n value determined using the Korsmeyer–Peppas model has previously been employed to determine the mechanisms by which drugs were released from various geometric matrices (e.g., slab, cylinder, and sphere) [69,70]. More specifically, for the drug release system of polymeric films, the diffusional exponent n was found to be equal to 0.5, be between 0.5 and 1.0, or to be equal to 1.0, indicating the diffusion mechanism to be Fickian diffusion, anomalous transport, or case-II transport, respectively. We show in Table 3 that, in this study, the n value for the drug release from each group was within the range of 0.5–1.0, suggesting that the mechanism was anomalous transport [69]. Our results indicated that the value of n for the drug release from the membrane with CMC:WPI-AF at 1:1 was found to be larger than that from the membrane with CMC:WPI-AF at 1:2.

Finally, the drug release results can be interpreted as indicating the following. First, as the CMC and WPI-AF were negatively charged at pH 7.4, they could electrostatically interact with the MB, leading to a large amount of drug absorption into the membrane. Conversely, when the MB interacted with the membranes through electrostatic interactions, it resulted in a diffusion barrier/resistance to drug release, leading to a lower drug release rate [65]. Second, increasing the WPI-AF content in the membranes rendered the membrane structure compact and increased the interaction between WPI-AF and drug, resulting in a significantly lower amount of drug release. Third, the MB and RF release mechanisms corresponded to non-Fickian transport controlled by diffusion and chain relaxation. In addition, the mechanism of drug release from the membranes was changed from being swelling controlled to being diffusion controlled by increasing the WPI-AF content in the membranes [42], suggesting different drug release behaviors to be presented by membranes with different CMC:WPI-AF mass ratios. These findings confirmed that CMC/WPI-AF membranes exhibit the potential to modulate the drug release rates and drug release behaviors of cationic (MB) and hydrophobic (RF) drugs through altering the CMC:WPI-AF mass ratio or WPI-AF content.

### 3.8. Discussion

Previous investigations have indicated that the polymeric vehicles (e.g., polyvinyl alcohol cryogel [71], polyethersulfone/polyacrylic acid composite hydrogel membrane [72], polyethylene oxide/pentaerythritol triacrylate/multi-walled carbon nanotubes composites [73], copolymer (PVA-co-PE) nanofiber membrane [74], and gelatin-polyacrylamide hydrogel [75]) have been commonly utilized in TDD systems. However, there are some potential problems/concerns that may arise when using the polymeric vehicles for drug delivery systems. These potential problems/concerns include cytotoxicity and lack of biocompatibility, generation of hazardous byproducts upon degradation, and usage of toxic additives or solvents during fabrication [76].

Amyloid fibrils exhibit remarkable properties, such as structural stability, mechanical stiffness, and abundance of functional groups [30]. Evidence has suggested that amyloid fibrils derived from food/milk proteins (e.g., whey, soybean, and egg white) show negligible cytotoxic effects toward human cells (Caco-2 and Hec-1a) in vitro [77], highlighting the biocompatible nature of food/milk protein-derived amyloid fibrils (e.g., WPI-AF). In addition, it was previously demonstrated that AFs derived from milk proteins (e.g., β-lactoglobulin) are able to enhance skin bio-adhesivity and anti-inflammatory activity of drugs used for topical treatments in vivo [78]. As a result, amyloid-based materials are suitable for a variety of biomedical applications, including drug delivery, biomineralization, and cell scaffolds [79]. Moreover, the above-mentioned potential problems and/or drawbacks associated with polymeric vehicles may be overcome when using amyloid-based materials (e.g., WPI-AF) as drug delivery carriers. Table S2 summarizes amyloid-based materials with different forms (e.g., fibrillar aggregates, membranes, and hydrogels) used in drug delivery systems. Application of amyloid-based materials have been explored in intravenous and gastrointestinal drug delivery systems, but very few reports have examined their usage in transdermal drug delivery (TDD) systems. To that end, we have prepared WPI amyloid-based membranes and tested the possibility of using the as-prepared WPI amyloid-based membranes for TDD systems in this study.

## 4. Conclusions

In summary, we have successfully synthesized two-dimensional carboxymethyl cellulose/whey protein isolate amyloid fibril (CMC/WPI-AF) membranes via chemical cross-linking coupled with phase inversion. The physiochemical features of these membranes were characterized and used as the delivery carriers for two different drugs (e.g., MB and RF). In addition, the influence of WPI-AF content on the aforesaid membranes’ zeta potential, surface microstructure, and drug encapsulation ability was examined. For the in vitro passive drug release process, the release rate and amount of total drug release were governed by the amount of WPI-AF and the binding affinity between the drug and WPI-AF. Moreover, our results demonstrated that the molecular mechanism by which the drugs released from CMC/WPI-AF membranes is governed by the combination of diffusion and chain relaxation, suggesting that the drug release behavior is mainly dependent upon the drug–membrane interaction and membrane properties. Furthermore, the release of drugs from CMC/WPI-AF membranes could be modulated by the WPI-AF content in the membrane. The outcome from this study highlights the potential of hybrid membranes made from protein amyloid fibrils for drug delivery applications.

## Figures and Tables

**Table 1:** A listing of the encapsulation efficiency and loading capacity of CMC/WPI-AF membranes on methylene blue and riboflavin.

CMC/WPI-AF Membranes	Methylene Blue (MB)	
	Encapsulation Efficiency (%)	Loading Capacity (%)
CMC:WPI-AF = 1:1	18.31 ± 3.14	106.71 ± 7.97
CMC:WPI-AF = 1:2	46.80 ± 2.88	197.38 ± 9.43
CMC/WPI-AF membranes	Riboflavin (RF)	
	Encapsulation efficiency (%)	Loading capacity (%)
CMC:WPI-AF = 1:1	10.74 ± 1.63	21.89 ± 1.21
CMC:WPI-AF = 1:2	16.49 ± 3.48	24.80 ± 1.49

**Table 2:** Estimates of the first order model parameters for MB or RF release from CMC/WPI-AF membranes synthesized at CMC:WPI-AF mass ratios of 1:1 and 1:2.

CMC/WPI-AF Membranes	Methylene Blue (MB)		
	M_∞_	k_f_ (min^−1^)	R^2^
CMC:WPI-AF = 1:1	54.35 ± 1.41	0.0111 ± 0.0005	0.996
CMC:WPI-AF = 1:2	21.66 ± 0.67	0.0127 ± 0.0008	0.993
CMC/WPI-AF membranes	Riboflavin (RF)		
	M_∞_	k_f_ (min^−1^)	R^2^
CMC:WPI-AF = 1:1	43.92 ± 1.19	0.4294 ± 0.0439	0.951
CMC:WPI-AF = 1:2	19.86 ± 0.53	0.3442 ± 0.0293	0.961

**Table 3:** Estimates of the Korsmeyer-Peppas model parameters for MB or RF release from CMC/WPI-AF membranes synthesized at CMC:WPI-AF mass ratios of 1:1 and 1:2.

CMC/WPI-AF Membranes	Methylene Blue (MB)		
	k_p_ (min^−n^)	N	R^2^
CMC:WPI-AF = 1:1	0.015 ± 0.002	0.852 ± 0.025	0.996
CMC:WPI-AF = 1:2	0.024 ± 0.005	0.755 ± 0.055	0.976
CMC/WPI-AF membranes	Riboflavin (RF)		
	k_p_ (min^−n^)	N	R^2^
CMC:WPI-AF = 1:1	0.326 ± 0.014	0.821 ± 0.057	0.992
CMC:WPI-AF = 1:2	0.249 ± 0.020	0.750 ± 0.090	0.973

## Data Availability

Can be provided by the authors upon request.

## Supplementary Materials

The following supporting information can be downloaded at: https://www.mdpi.com/article/10.3390/polym15061444/s1, Table S1: A listing of composition and key properties of whey protein isolate (WPI) used in this study; Table S2: A summary of amyloid-based materials used for drug delivery; Figure S1: The chemical structure of (A) methylene blue and (B) riboflavin; Figure S2: (A) The UV-Vis spectra of methylene blue with different concentrations. (B) The UV-Vis spectra of riboflavin with different concentrations. (C) The calibration curve of methylene blue. (D) The calibration curve of riboflavin. The absorbance of methylene blue and riboflavin was measured at the wavelength of 665 nm and 445 nm, respectively [42,80,81,82,83,84,85,86,87,88].
