# Supplementary material for: Application of Amyloid-Based Hybrid Membranes in Drug Delivery
Source: Polymers (Basel). 2023 Mar 14;15(6):1444. doi: 10.3390/polym15061444 (PMC10052896; doi:10.3390/polym15061444)
Supplement: Supplementary file 1 [file polymers-15-01444-s001.zip › polymers-2237435-supplementary.pdf]

polymers

Supplementary Materials

Application of Amyloid-Based Hybrid Membranes in Drug Delivery

You-Ren Lai [1,†], Steven S.-S. Wang [1,†], Ti-Lun Hsu [1], Szu-Hui Chou [1], Su-Chun How [2,*] and Ta-Hsien Lin [3,4,*]

[1] Department of Chemical Engineering, National Taiwan University, Taipei 10617, Taiwan; ray110135@gmail.com (Y.-R.L.); sswang@ntu.edu.tw (S.S.-S.W.)
[2] Department of Chemical Engineering and Biotechnology, Tatung University, Taipei 104, Taiwan
[3] Laboratory of Nuclear Magnetic Resonance, Department of Medical Research, Taipei Veterans General Hospital, Taipei 11217, Taiwan
[4] Institute of Biochemistry and Molecular Biology, National Yang Ming Chiao Tung University, Taipei 11221, Taiwan
* Correspondence: schow@gm.ttu.edu.tw (S.-C.H.); thlin@vghtpe.gov.tw (T.-H.L.); Tel.: +886-2-2182-2928 (ext. 6216) (S.-C.H.); +886-2-2871-2121 (ext. 2703) (T.-H.L.)
† These authors contributed equally to this work.

Table S1. A listing of composition and key properties of whey protein isolate (WPI) used in this study.

Protein	β-lactoglobulin (β-LG)	α-lactalbumin (α-LA)	Bovine serum albumin (BSA)
**Composition**	~48-58 %	~13-19 %	~6 %
**PDB structure**	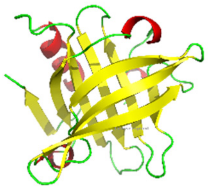	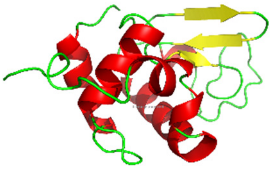	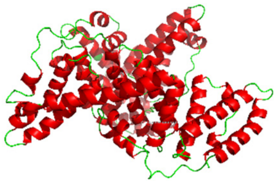
**Number of residue**	162	123	582
**Molecular weight**	~18.3 kDa	~14.2 kDa	~66.4 kDa
**Number of thiol group and disulfide bond**	1 free thiol group and 2 S-S bonds	4 S-S bonds	1 free thiol group and 17 S-S bonds
**Isoelectric point (pI)**	~5.2	~4.8	~4.7

Table S2. A summary of amyloid-based materials used for drug delivery.

Materials	Drug delivery vehicles	Fabrication techniques	Drug types	References
β-LG fibril	Fibrous aggregates	Fibrillization process	PdNP, AuNP, AgNP	[1]
β-LG fibril	Fibrous aggregates	Fibrillization process	Iron nanoparticle	[2]
GA/WPI-AF	Fibrous aggregates	Fibrillization process	Iron ions (Fe^{3+})	[3]
Lysozyme fibril	Microgel	Droplet microfluidics	ThT, RBBR, Penicillin V, Tetracycline	[4]
β-LG/BSA fibril	Hydrogel	Ca^{2+}-induced cold-set gelation	Riboflavin	[5]
Lysozyme fibril	Hydrogel	Heat-induced gelation	Beta-blokers	[6]
αS fibril	Hydrogel	Disulfide-exchange process	Rhodamine 6G	[7]
Lysozyme fibril	Injectable hydrogel	Physical gelation	Doxorubicin	[8]
MAX8 peptide fibril	Injectable hydrogel	Physical gelation	Curcumin	[9]
BSA fibril	Membrane	Electrospinning	Ampicillin	[10]
CMC/WPI-AF	Membrane	Chemical crosslink/Phase inversion	Methylene blue, Riboflavin	Present work

Abbreviations: WPI-AF – whey protein isolate amyloid fibril; CMC – carboxymethyl cellulose; β-LG – β-lactoglobulin; BSA – bovine serum albumin; αS – α-synuclein; GA – gum arabic; PdNP – palladium nanoparticle; AgNP – silver nanoparticle ; AuNP – gold nanoparticle; Beta-blokers – beta-adrenoceptor antagonists; ThT – thioflavin T; RBBR – remazol brilliant blue R.

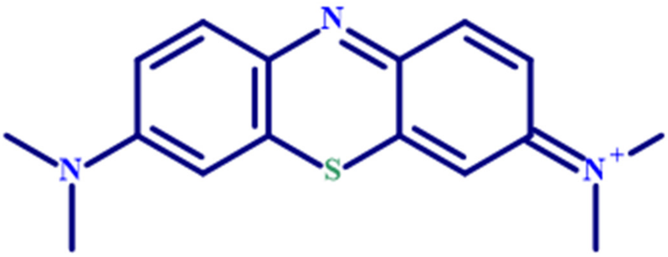

(**A**)

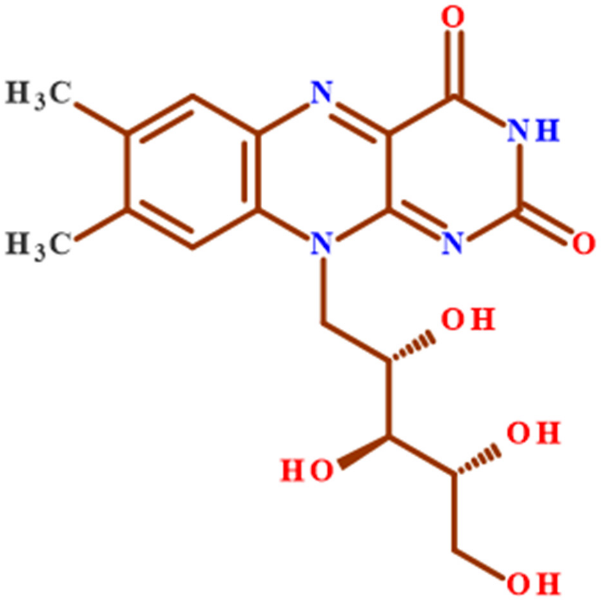

(**B**)

Figure S1. The chemical structure of (A) methylene blue and (B) riboflavin.

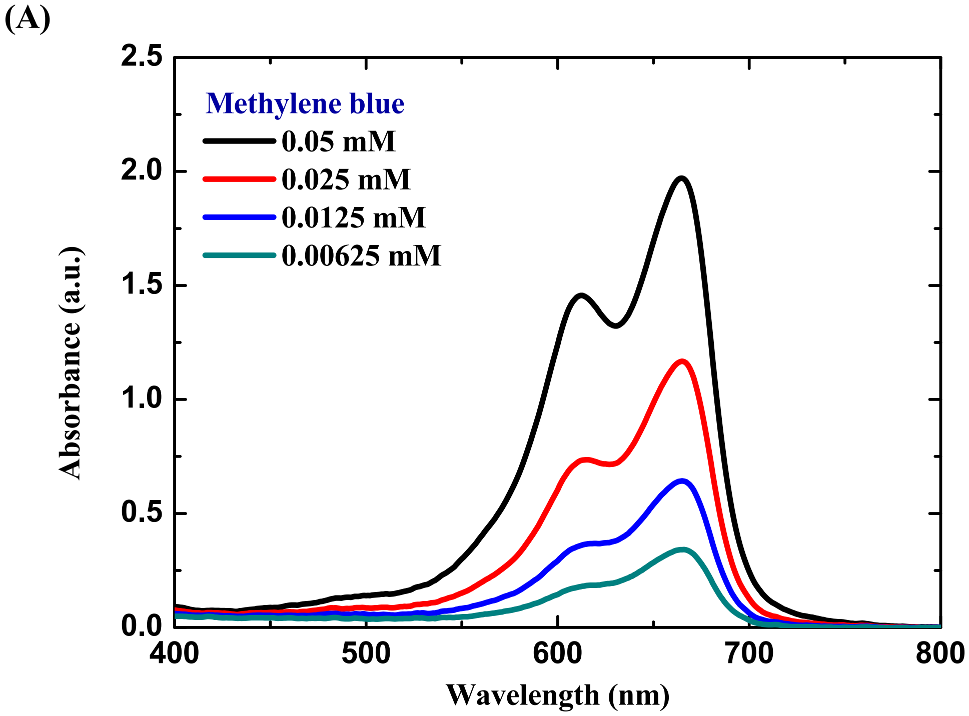

(B)

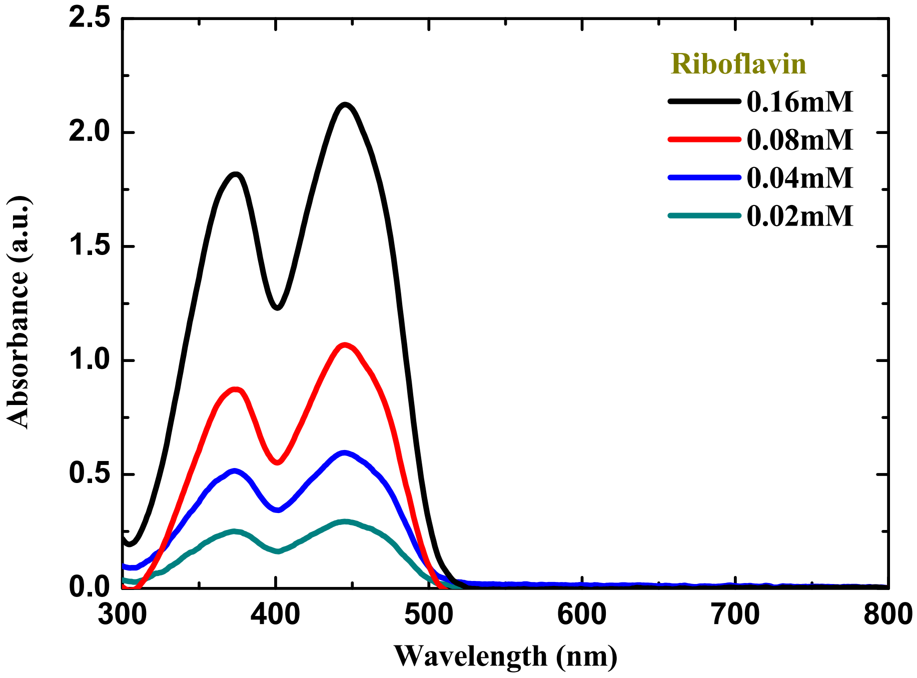
Riboflavin
0.16mM
0.08mM
0.04mM
0.02mM
Absorbance (a.u.)
Wavelength (nm)
2.5
2.0
1.5
1.0
0.5
0.0
300
400
500
600
700
800

(C)

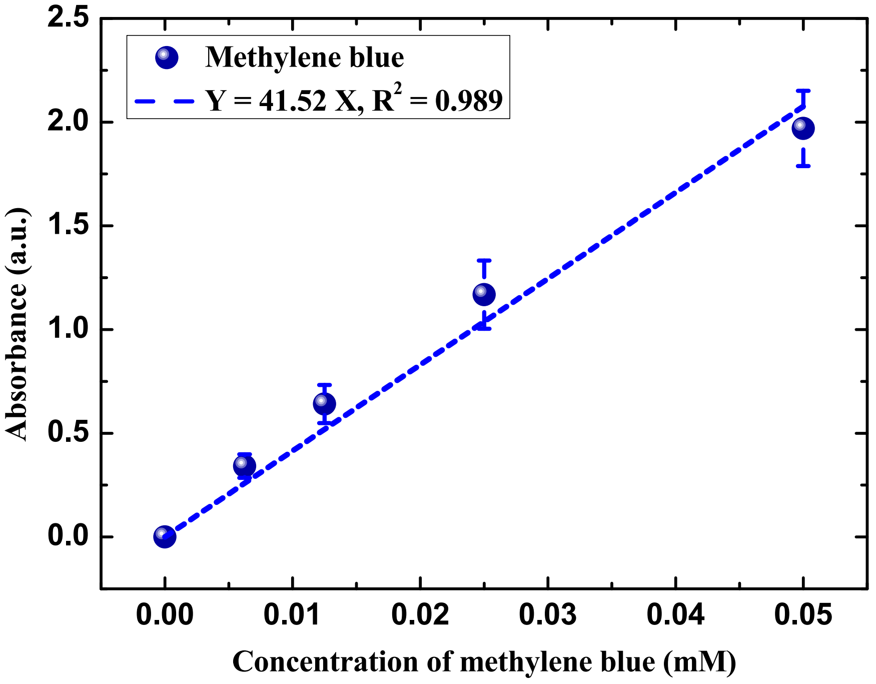
Methylene blue
Y = 41.52 X, R2 = 0.989
Absorbance (a.u.)
Concentration of methylene blue (mM)
2.5
2.0
1.5
1.0
0.5
0.0
0.00
0.01
0.02
0.03
0.04
0.05

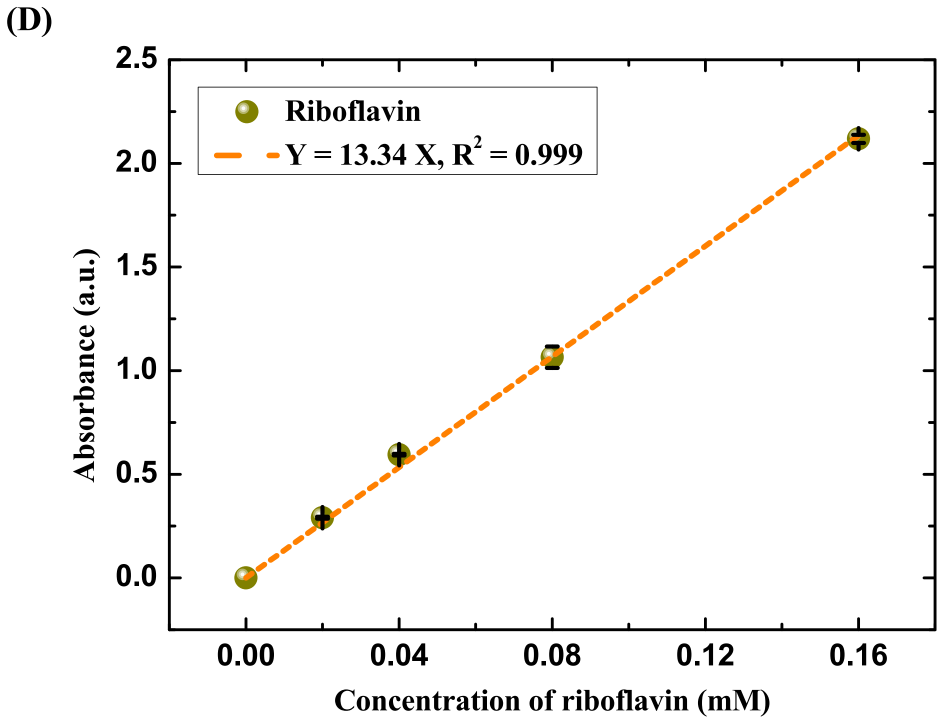

Figure S2. (A) The UV-Vis spectra of methylene blue with different concentrations. (B) The UV-Vis spectra of riboflavin with different concentrations. (C) The calibration curve of methylene blue. (D) The calibration curve of riboflavin. The absorbance of methylene blue and riboflavin was measured at the wavelength of 665 nm and 445 nm, respectively.

References

1. Bolisetty, S.; Boddupalli, C.S.; Handschin, S.; Chaitanya, K.; Adamcik, J.; Saito, Y.; Manz, M.G.; Mezzenga, R. Amyloid fibrils enhance transport of metal nanoparticles in living cells and induced cytotoxicity. *Biomacromolecules* **2014**, *15*, 2793–2799.
2. Shen, Y.; Posavec, L.; Bolisetty, S.; Hilty, F.M.; Nyström, G.; Kohlbrecher, J.; Hilbe, M.; Rossi, A.; Baumgartner, J.; Zimmermann, M.B. Amyloid fibril systems reduce, stabilize and deliver bioavailable nanosized iron. *Nat. Nanotechnol.* **2017**, *12*, 642–647.
3. Yue, J.; Shu, M.; Yao, X.; Chen, X.; Li, D.; Yang, D.; Liu, N.; Nishinari, K.; Jiang, F. Fibrillar assembly of whey protein isolate and gum Arabic as iron carrier for food fortification. *Food Hydrocoll.* **2022**, *128*, 107608.
4. Shimanovich, U.; Efimov, I.; Mason, T.O.; Flagmeier, P.; Buell, A.K.; Gedanken, A.; Linse, S.; Åkerfeldt, K.S.; Dobson, C.M.; Weitz, D.A. Protein microgels from amyloid fibril networks. *ACS nano* **2015**, *9*, 43–51.
5. How, S.-C.; Lin, T.-H.; Chang, C.-C.; Wang, S.S.-S. Examining the effect of bovine serum albumin on the properties and drug release behavior of β-lactoglobulin-derived amyloid fibril-based hydrogels. *Int. J. Biol. Macromol.* **2021**, *184*, 79–91.
6. Mains, J.; Lamprou, D.A.; McIntosh, L.; Oswald, I.D.; Urquhart, A.J. Beta-adrenoceptor antagonists affect amyloid nanostructure; amyloid hydrogels as drug delivery vehicles. *Chem. Commun.* **2013**, *49*, 5082–5084.
7. Ha, Y.; Kwon, Y.; Nam, E.-J.; Park, H.; Paik, S.R. Disulfide-mediated elongation of amyloid fibrils of α-synuclein for use in producing self-healing hydrogel and dye-absorbing aerogel. *Acta Biomater.* **2022**, *145*, 52–61.
8. Yang, L.; Li, H.; Yao, L.; Yu, Y.; Ma, G. Amyloid-based injectable hydrogel derived from hydrolyzed hen egg white lysozyme. *Acs Omega* **2019**, *4*, 8071–8080.
9. Altunbas, A.; Lee, S.J.; Rajasekaran, S.A.; Schneider, J.P.; Pochan, D.J. Encapsulation of curcumin in self-assembling peptide hydrogels as injectable drug delivery vehicles. *Biomaterials* **2011**, *32*, 5906–5914.
10. Kabay, G.; Meydan, A.E.; Can, G.K.; Demirci, C.; Mutlu, M. Controlled release of a hydrophilic drug from electrospun amyloid-like protein blend nanofibers. *Mater. Sci. Eng. C* **2017**, *81*, 271–279.
